# A Hypomorphic *Dars1*^*D*367*Y*^ Model Recapitulates Key Aspects of the Leukodystrophy HBSL

**DOI:** 10.3389/fncel.2020.625879

**Published:** 2021-01-20

**Authors:** Dominik Fröhlich, Marisa I. Mendes, Andrew J. Kueh, Andre Bongers, Marco J. Herold, Gajja S. Salomons, Gary D. Housley, Matthias Klugmann

**Affiliations:** ^1^Translational Neuroscience Facility & Department of Physiology, School of Medical Sciences, UNSW Sydney, Kensington, NSW, Australia; ^2^Metabolic Unit/Laboratory Genetic Metabolic Diseases, Department of Clinical Chemistry, Amsterdam Neuroscience, Amsterdam Gastroenterology & Metabolism, Amsterdam UMC, Vrije Universiteit Amsterdam, Amsterdam, Netherlands; ^3^The Walter and Eliza Hall Institute of Medical Research, Parkville, VIC, Australia; ^4^Department of Medical Biology, University of Melbourne, Parkville, VIC, Australia; ^5^Biomedical Resources Imaging Laboratory, UNSW Sydney, Kensington, NSW, Australia

**Keywords:** HBSL, DARS1, AspRS, aminoacyl-tRNA synthetase, aspartyl-tRNA synthetase, leukodystrophy

## Abstract

Hypomyelination with brain stem and spinal cord involvement and leg spasticity (HBSL) is a leukodystrophy caused by missense mutations of the aspartyl-tRNA synthetase-encoding gene *DARS1*. The clinical picture includes the regression of acquired motor milestones, spasticity, ataxia, seizures, nystagmus, and intellectual disabilities. Morphologically, HBSL is characterized by a distinct pattern of hypomyelination in the central nervous system including the anterior brainstem, the cerebellar peduncles and the supratentorial white matter as well as the dorsal columns and the lateral corticospinal tracts of the spinal cord. Adequate HBSL animal models are lacking. *Dars1* knockout mice are embryonic lethal precluding examination of the etiology. To address this, we introduced the HBSL-causing *Dars1*^*D*367*Y*^ point mutation into the mouse genome. Surprisingly, mice carrying this mutation homozygously were phenotypically normal. As hypomorphic mutations are more severe in trans to a deletion, we crossed *Dars1*^*D*367*Y*/*D*367*Y*^ mice with *Dars1-null* carriers. The resulting *Dars1*^*D*367*Y*/−^ offspring displayed a strong developmental delay compared to control *Dars1*^*D*367*Y*/+^ littermates, starting during embryogenesis. Only a small fraction of *Dars1*^*D*367*Y*/−^ mice were born, and half of these mice died with hydrocephalus during the first 3 weeks of life. Of the few *Dars1*^*D*367*Y*/−^ mice that were born at term, 25% displayed microphthalmia. Throughout postnatal life, *Dars1*^*D*367*Y*/−^ mice remained smaller and lighter than their *Dars1*^*D*367*Y*/+^ littermates. Despite this early developmental deficit, once they made it through early adolescence *Dars1*^*D*367*Y*/−^ mice were phenotypically inconspicuous for most of their adult life, until they developed late onset motor deficits as well as vacuolization and demyelination of the spinal cord white matter. Expression levels of the major myelin proteins were reduced in *Dars1*^*D*367*Y*/−^ mice compared to controls. Taken together, *Dars1*^*D*367*Y*/−^ mice model aspects of the clinical picture of the corresponding missense mutation in HBSL. This model will enable studies of late onset deficits, which is precluded in *Dars1* knockout mice, and can be leveraged to test potential HBSL therapeutics including *DARS1* gene replacement therapy.

## Introduction

Leukodystrophies are inherited white matter disorders often associated with an early onset, lack of treatment options and premature death. The population incidence of all leukodystrophies taken together is relatively high with one in 7,600 live births (Bonkowsky et al., 2010) underpinning the high unmet medical need. *H*ypomyelination with *b*rain stem and *s*pinal cord involvement and *l*eg spasticity (HBSL) belongs to this group of diseases and was first described in 2013 (Taft et al., 2013). Following the initial discovery, two additional case studies, together with the original study, described a total of 16 HBSL patients (Wolf et al., 2015; Ong et al., 2020). HBSL can be seen as a spectrum disorder with a high variance in severity (mild to severe forms) and onset of the disease (4 months to 22 years) (Wolf et al., 2015). An early onset form of HBSL usually results in a more severe course of disease. The clinical symptoms typically include motor deficits, leg spasticity, regression, or delay of developmental milestones, hypertonia, hyperreflexia, positive Babinski sign, nystagmus, and gait abnormalities in patients who are able to mobilize (Taft et al., 2013; Wolf et al., 2015; Ong et al., 2020). For a comprehensive clinical review of HBSL see Muthiah et al. (2020) in this issue.

The underlying cause of HBSL are missense mutations of the aspartyl-tRNA synthetase (AspRS) gene *DARS1*. AspRS belongs to a group of enzymes termed aminoacyl-tRNA synthetases (ARSs) that catalyze an aminoacylation reaction in which transfer ribonucleic acids (tRNAs) are linked to their cognate amino acids. This process is known as tRNA charging and is an essential prerequisite for successful protein biosynthesis. Each ARS is specific for the charging of one tRNA with its corresponding amino acid, e.g., AspRS specifically links tRNA^Asp^ to aspartate, and there is no redundancy amongst these enzymes. ARSs can be subdivided into two classes depending on the cell compartment in which they catalyze the aminoacylation reaction: Cytosolic ARSs and mitochondrial ARSs (mt-ARSs). AspRS functions as a homodimer (Kim et al., 2013) and is ubiquitously expressed in all cells and tissues. However, our previous studies revealed a strong prevalence in neurons of the cerebellum in the murine (Fröhlich et al., 2017) and human brain (Fröhlich et al., 2018). All *DARS1* mutations identified to date are located within the catalytic domain of AspRS and are likely having a direct impact on the aminoacylation reaction and consequently on protein synthesis. Why *DARS1* mutations specifically manifest in neurologic deficits remains unresolved.

Previous attempts at creating a mouse model for HBSL through complete knockout of the *Dars1* gene were unsuccessful and either resulted in early embryonic lethality (homozygous *Dars1*-null mice), or the lack of HBSL pathology (heterozygous *Dars1*-null mice). A more promising strategy is to introduce HBSL-causing *DARS1* point mutations into the mouse gene. The HBSL index patient was reported to be a compound heterozygous carrier of the *DARS1*^*A*274*V*^ and *DARS1*^*D*367*Y*^ point mutations. In this study, we employed CRISPR/Cas9-mediated gene editing to introduce the *Dars1*^*D*367*Y*^ mutation into the murine *Dars1* gene. Surprisingly, homozygous *Dars1*^*D*367*Y*/*D*367*Y*^ mice were only mildly affected. The human *DARS1* missense mutations identified so far can be classified as hypomorphic mutations, which means that they cause a partial loss of gene function either through reduced expression or through impaired activity. Hypomorphs do not usually result in a complete loss of function and are always more severe in trans to a deletion mutation. In order to enhance the phenotype of *Dars1*^*D*367*Y*/*D*367*Y*^ mice, we bred them with *Dars1-null* carriers. The *Dars1*^*D*367*Y*/−^ offspring displayed a strong developmental delay, which often resulted in early embryonic or pre-weaning death. The few surviving *Dars1*^*D*367*Y*/−^ mice were smaller and lighter compared to *Dars1*^*D*367*Y*/+^ littermates and developed late onset motor deficits as well as vacuolization of the white matter of the spinal cord. In summary, *Dars1*^*D*367*Y*/−^ mice recapitulate some HBSL aspects and will be instrumental for examinations of the disease etiology or therapeutic proof-of-concept studies.

## Methods

### Ethics

All procedures were approved by the University of New South Wales Animal Care and Ethics Committee and were conducted in accordance with the Australian Code of Practice for the Care and Use of Animals for Scientific Purposes.

### Animals

Mice were housed in ventilated cages in groups of 2 to 5 animals and fed *ad libitum* with standard chow. The c.1099G>T (D367Y) point mutation was introduced into the *Dars1* locus using the CRISPR/Cas9 technique as previously described (Kueh et al., 2017) on a C57BL/6J background. To generate mice carrying the *Dars1* c.1099G>T (D367Y) point mutation, 20 ng/μl of Cas9 mRNA, 10 ng/μl of sgRNA (CGATGAGGAAGATCTAAGGT) and 40 ng/μl of oligo donor (gagtattgcgaggctttggcaatgcttagagaagctggagttgaaatggacgatgaggaaTatctaaggttAgtctctgatattttctctttcaactttaatgcttaggtttaatgtatttcacccagctga) were injected into the cytoplasm of fertilized one-cell stage embryos. Twenty-four hours later, two-cell stage embryos were transferred into the oviducts of pseudo-pregnant female mice. Viable offspring were genotyped by next-generation sequencing. Additionally, correct targeting was also confirmed by Sanger sequencing. The region containing the c.1099G>T mutation was amplified by PCR using the following primers: Forward 5′-GCTTTTCTTGGTTCAGTCGC-3′, reverse 5′-CGGGTTACAACTAGGGGAAA-3′. The resulting 449 bp PCR fragment was purified by ethanol precipitation and subsequently sequenced using the forward primer.

*Dars1*-null mice were created and genotyped as previously described (Fröhlich et al., 2017). *Dars1*^*D*367*Y*/−^ mice were generated by breeding homozygous *Dars1*^*D*367*Y*/*D*367*Y*^ mice with heterozygous *Dars1*-null mice.

### Behavioral Testing

Behavioral tests were either performed in *Dars1*^*D*367*Y*/*D*367*Y*^ mice together with age- and sex-matched wildtype mice or in age- and sex-matched *Dars1*^*D*367*Y*/−^ and *Dars1*^*D*367*Y*/+^ littermates. Locomotor behavior was assessed using the rotarod test as described (von Jonquieres et al., 2018). For habituation, mice were placed on the rotarod apparatus (Ugo Basile, Comerio, Italy) for 1 min at a constant speed of 4 revolutions per minute (rpm) before the test trials started. During the test, the speed of the rotarod constantly increased from 4 to 40 rpm over a 4-min period. Cut-off time was 5 min with the last min on full speed. Mice were tested in 3 trials per day on two consecutive days (6 trials in total). Inter-trial interval was 30 min. The latency to fall was averaged over the six trials.

The open field-test was performed as described (Fröhlich et al., 2017). Mice were placed in the center of an open field box (40 × 40 × 40 cm^3^) under bright light conditions (100 lux). Mice were video recorded during the 30 min trial and subsequently analyzed using the ANY-Maze™ tracking software (Stoelting, Illinnois, USA) for total distance traveled as well as the distance traveled in the inner compartment.

To determine the acoustic startle response (ASR) as well as the pre-pulse inhibition (PPI) we used the SR-LAB Startle Response System (San Diego Instruments, San Diego, USA) as described (Schneider et al., 2007; Fröhlich et al., 2017). During ASR and PPI, mice were exposed to 60 dB sound pressure level (SPL) background white noise and both the ASR and PPI, were preceded by a 5 min habituation period of 60 dB SPL white noise only. The ASR was measured as the maximum amplitude detected by the accelerometer in response to 40 ms white noise pulses with increasing intensities (60–120 dB SPL) with a 10 s interval. In order to determine the PPI, the 120 dB SPL startle pulse was preceded (100 ms) by a 20 ms pre-pulse (72, 76, or 80 dB SPL). Each trial was repeated 10 times. PPI was determined as the decrease of the ASR amplitude in response to the pre-pulse compared to the 120 dB SPL pulse alone.

### Magnetic Resonance Imaging (MRI) and Body Composition Analysis

Following a lethal intraperitoneal (IP) injection of pentobarbital, mice were transcardially perfused with 10 ml phosphate buffered saline (PBS) for 5 min, followed by 10 ml 4% paraformaldehyde (PFA) for 5 min. Brains were post-fixed in 4% PFA for 2 h at room temperature (RT). Prior to imaging the fixed brains were immersed in in 9 g/l NaCl/H_2_O solution for 24 h at 21°C to remove fixation residues in the brain tissue. The brains were then transferred into a 1.3 mm ID, 2 ml Cryovial (Greiner, Germany) and submersed in Perfluoro-Polyether Fomblin™ 6Y for susceptibility matching. The Cryovial was then mounted on the tip of a plastic tube, which was attached to the automatic positioning system of the MRI system. MRI was performed using a 9.4T BioSpec Avance III 94/20 (Bruker, Ettlingen, Germany) magnetic resonance microimaging system equipped with BGA-12S HP gradients with maximum strength 660 mT/m and slew rate 4,570 Tm/s. A dedicated 15 mm internal diameter quadrature specimen volume coil was used for radiofrequency transmission and reception. Anatomical images were acquired using an optimized isotropic 3D multi gradient echo sequence (MGE) with 106 coronal partitions and 10 gradient echoes with the following major parameters: First TE = 2.7 ms, ΔTE = 3.45 ms, #echos = 28, TR = 100 ms, FA = 30°, FOV = 15 × 15 × 8 mm, matrix = 200 × 200× 106, image resolution = 75 μm^3^ (isotropic), eff. spectral BW = 78,125 Hz, total acquisition time with 2 ADC averages: 1 h and 46 min per specimen. Segmentation and surface models of the entire brain as well as the ventricles were generated using the 3D slicer software (Kikinis et al., 2014).

Body composition analysis (EchoMRI) was performed as described (von Jonquieres et al., 2018) in accordance to the manufacturer's instructions using the EchoMRI-900^TM^ scanner equipped with A100 mouse antenna insert (Echo Medical Systems). Fat and lean mass were calculated as percentage of total mass.

### Mouse Histopathology Evaluation

This study utilized the Phenomics Australia (PA; formerly known as Australian Phenomics Network) Histopathology and Organ Pathology Service (HOPS) at the University of Melbourne. This service included: Harvesting of 25 organs, fixation and embedding in paraffin blocks, sectioning, Haematoxylin and Eosin (H&E; all organs; **Figures 3H,I**, **5C**) and Luxol Fast Blue (LFB; brain and spinal cord; **Figures 5B,D**) staining, slide scanning, images of histopathology, and detailed histology and pathology reports.

### Immunohistochemistry

Following cardiac perfusion as described above, brains were post-fixed in 4% PFA for 2 h at RT and cryoprotected in 30% sucrose. Brains were cut in the coronal plane into 40 μm sections using a cryostat as described (von Jonquieres et al., 2013). Sections were permeabilized with 0.2% TritonX-100 in PBS (PBS-Tx) and non-specific binding was blocked with 4% goat serum in PBS-Tx. Primary antibodies were applied in 4% goat serum in PBS-Tx over night at 4°C. The following antibodies were used: rabbit anti-NF200 (neurofilament 200; 1:1000; Sigma-Aldrich no. N4142) and rat anti-PLP clone aa3 (proteolipid protein; 1:10; gift from Prof. J. Trotter, Mainz, Germany). After washing with PBS, sections were incubated with goat anti-rabbit Alexa-488 and goat anti-rat Alexa-594 secondary antibodies (1:400; Thermo Fisher Scientific no. A11012 and A11006) in 4% goat serum in PBS-Tx for 4 h at RT. After washing in PBS-Tx, sections were mounted in Mowiol (Calbiochem, Darmstadt, Germany) and imaged using an LSM710 confocal microscope (Carl Zeiss, Berlin, Germany).

### RNA Isolation and qRT-PCR

Animals were euthanized at 10 months by cervical dislocation. The brain regions cortex (CX), cerebellum (CB), brainstem (BS), and basal ganglia (BG) were dissected and snap frozen. RNA extraction was performed as described before (Fröhlich et al., 2018). Briefly, brain tissue was homogenized in liquid nitrogen using mortar and pestle followed by RNA extraction according to the manufacturer's instructions (RNeasy MiniKit, Qiagen no. 74106) including on-column DNase digest (RNase-Free DNase Set, Qiagen no. 79254). RNA was transcribed into cDNA using the High Capacity cDNA Reverse Transcription Kit (Applied Biosystems no. 4368813) according to the manufacturer's instructions. Quantitative real-time PCR (qRT-PCR) was performed in triplicate using the StepOnePlus™ Real-Time PCR system (Applied Biosystems). The following TaqMan probes (Applied Biosystems) were used: *Dars1* (Mm00624185_m1), *Dars2* (Mm01296063_m1), *Aspa* (Mm004808667_m1), *Plp1* (Mm00456892_m1), *Cnp* (Mm01306640), *Mbp* (Mm01266402_m1), *GusB* (Mm01197698_m1). Data was normalized to the housekeeper *GusB* and the CX region using the comparative ΔΔCT method.

### SDS-PAGE and Western-Blotting

Animals were euthanized at 10 months by cervical dislocation. The brain regions CX, CB, BS, and BG were dissected, snap frozen and homogenized under liquid nitrogen using mortar and pestle. 10 μl lysis buffer (50 mM Tris-Cl, pH 7.4, 1 mM EDTA pH 8.0, 250 mM NaCl, and 1% Triton-X) including a cocktail of protease inhibitors (Complete, Roche) were added per mg of brain tissue. Lysates were sonicated using a Branson 450 Digital Probe Sonifier at 10% sonication amplitude and protein concentration was determined by Bradford protein assay (Bio-Rad no. 5000006).

SDS-PAGE and Western-blotting was performed as described (Fröhlich et al., 2018). In summary, 20 μg of protein mixed with 5x Laemmli reducing sample buffer were loaded onto a 10% acrylamide gel, separated by SDS-PAGE, and transferred onto a PVDF membrane (Bio-Rad no. 162-0177). Membranes were incubated with 4% skim milk powder in PBS plus 0.1% Tween (PBS-T) to prevent unspecific binding of antibodies. Subsequently, membranes were probed with the following primary antibodies in 4% skim milk in PBS-T: mouse anti-AspRS (1:1000; SantaCruz no. sc-393275), rabbit anti-GAPDH (glyceraldehyde 3-phosphate dehydrogenase; 1:4000, Cell Signaling no. 2118S), mouse anti-CNP (2',3'-cyclic nucleotide 3' phosphodiesterase; 1:3000, Abcam no. ab6319), rat anti-MBP (myelin basic protein; 1:1000; Abcam no. ab7349), rat anti-PLP aa3 (proteolipid protein; 1:200, gift from Prof. J. Trotter, Mainz, Germany). Following 3 wash steps (15 min each) with PBS-T, membranes were probed with appropriate HRP-conjugated secondary antibodies (1:10000; Dianova, Hamburg, Germany) in 4% skim milk in PBS-T. After 3 additional wash steps with PBS-T, membranes were incubated for 1 min with 1 ml Clarity Western ECL substrate (Bio-Rad no. 170-5060) and imaged in the ChemiDoc MP system (Bio-Rad, Hercules, USA).

### AspRS Enzyme Activity Assay

HEK293 cells were cultured to 70% confluency for transient expression with *DARS1*^*WT*^, *DARS1*^*D*367*Y*^, *DARS1*^*A*274*V*^, or an equimolar mix of *DARS1*^*A*274*V*^ and *DARS1*^*D*367*Y*^. For transfection, 12 μg of plasmid DNA were mixed with 60 μl Fugene (Promega, Madison, WI) in serum–free DMEM medium (Thermo Fisher Scientific, Waltham, USA) and incubated for 15 min at room temperature. Subsequently, the mix was applied to the cells. Cells transfected with an empty vector (mock transfected) and untransfected cells were included as controls. Fluorescent microscopy was used to estimate transfection efficiency and Western blotting was performed to confirm comparable expression levels of wildtype and mutant AspRS. Cells were harvested 48 h post transfection, pelleted, snap-frozen, and stored at −80°C until further use. All transfections were performed in triplicate.

To determine aminoacylation activity, pellets were suspended in ddH_2_O and cells were lysed in three freeze-thaw cycles. Protein amount was quantified using the BCA assay and protein samples were adjusted to 1 mg/ml. Cell lysates were incubated in triplicate for 10 min at 37°C in reaction buffer [50 mM Tris buffer pH 7.5, 12 mM MgCl2, 25 mM KCl, 1 mg/ml bovine serum albumin, 0.5 mM spermine, 1 mM ATP, 0.2 mM *E. coli* total tRNA, 1 mM dithiothreitol, and 0.3 mM (^13^C_4_, ^15^N)-aspartate]. The reaction was stopped using trichloroacetic acid. After washing the samples with trichloroacetic acid, ammonia was added to release the labeled amino acids from the tRNAs. [D3]-aspartate was added as internal standard and the labeled amino acids were quantified by LC-MS/MS. Aminoacylation activity measured for mock transfected cells was subtracted from *DARS1*-transfected samples. *DARS1*^*WT*^ transfected cells were used as a positive control and activities were expressed as percentage of *DARS1*^*WT*^ transfected cells.

### Statistics

Statistical analysis was performed with the GraphPad Prism 8 software (La Jolla, CA, USA). Following validation of normal distribution of data, one-way or two-way analysis of variance (ANOVA) with Bonferroni's multiple comparisons *post-hoc* test was performed as indicated. Values are displayed as mean ± SEM with *p* < 0.05 regarded as statistically significant.

## Results

### Phenotype of Homozygous *Dars1^*D*367*Y*/*D*367*Y*^* Mice

**Figure 1:**
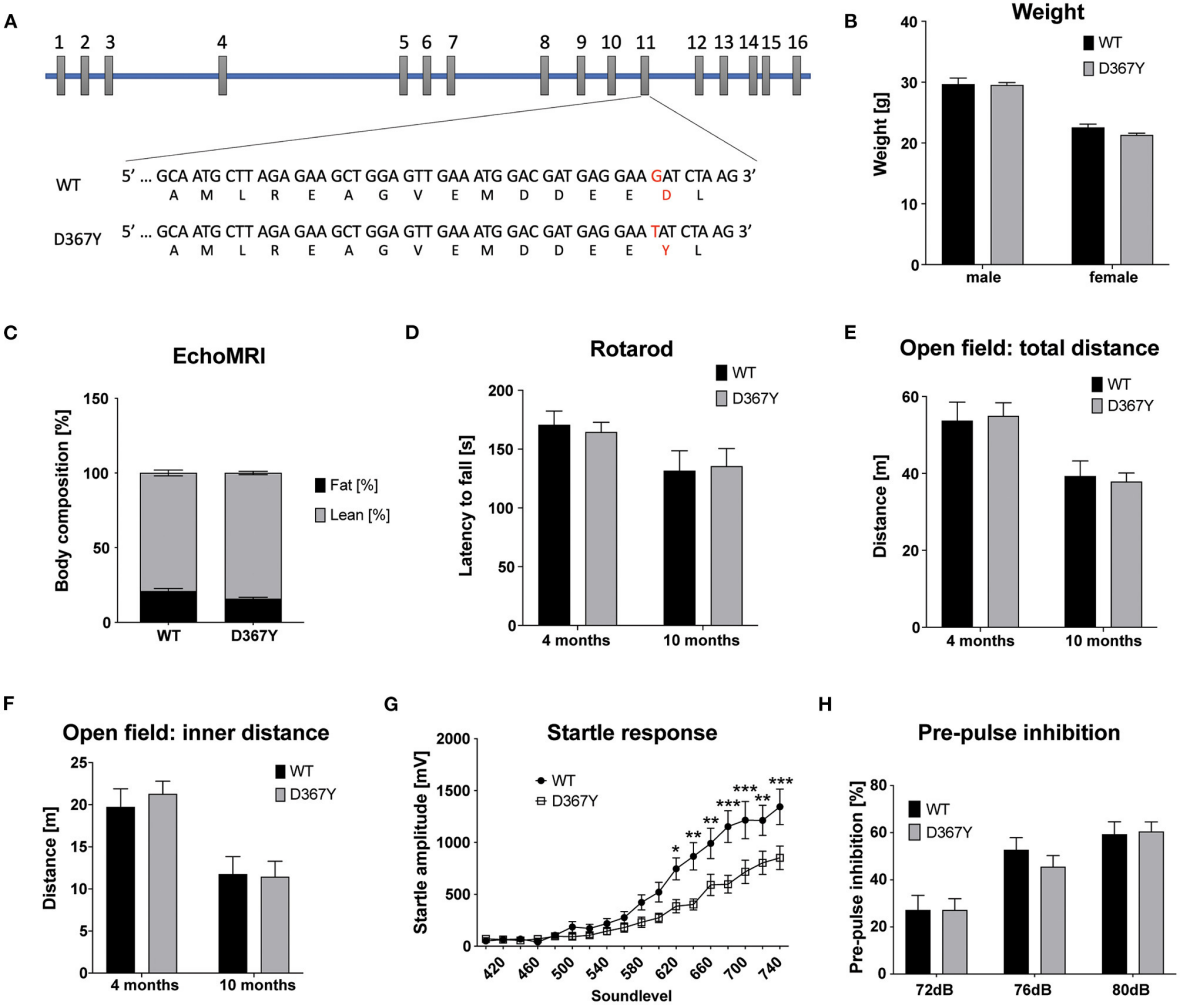
Characterization of homozygous *Dars1*^*D*367*Y*^ mice. **(A)** Schematic depicting the genomic location of the D367Y mutation on exon 11 of the Dars1 gene. **(B)** Body weight of homozygous D367Y mice compared to wildtype controls (WT) at 4 months of age (*n* = 4–6). **(C)** EchoMRI body composition analysis revealed a slight shift from fat to lean mass in homozygous D367Y mice (*n* = 9). **(D)** Motor coordination assessed by the rotarod test was unchanged between homozygous D367Y mice and wildtype controls (*n* = 8–24). **(E,F)** Total distance traveled in an open field test apparatus [**(E)**; n = 7–28] and distance in the inner compartment of the apparatus [**(F)**; *n* = 7–28]. **(G)** Four-months-old homozygous D367Y mice exposed to sound pulses with increasing intensities (60–120 dB SPL, 40 ms) showed a lowered acoustic startle response compared to wildtype controls (*n* = 33–38). **(H)** Pre-pulse inhibition (72, 76, or 80 dB SPL pre-pulse 100 ms before the 120 dB SPL startle pulse) was unchanged between genotypes (*n* = 19–36). Data represent mean ± SEM (*p < 0.05, **p < 0.01, ***p < 0.001; Two-way ANOVA).

In our previous study we demonstrated that the early embryonic lethality of *Dars1*-null mice precluded their use as an accurate model for HBSL (Fröhlich et al., 2017). In this study we introduced the c.1099G>T (D367Y) point mutation, which was first identified in the compound heterozygous index patient, into the mouse *Dars1* locus using the CRISPR/Cas9 gene editing technology. This single nucleotide change located on exon 11 of the *Dars1* locus results in a TAT codon coding for tyrosine instead of GAT coding for aspartic acid (Figure 1A). To our surprise, homozygous *Dars1*^*D*367*Y*/*D*367*Y*^ mice were only mildly affected. The body weight of homozygous *Dars1*^*D*367*Y*/*D*367*Y*^ mice, compared to wildtype controls at 4 months of age, was unchanged (Figure 1B). Body composition analysis using EchoMRI revealed only a slight shift from fat to lean mass without reaching statistical significance (Figure 1C). We performed behavioral tests with these mice at two different timepoints, at 4 and 10 months, to detect both early as well as late onset deficits. Motor coordination was assessed using the rotarod test but revealed no differences between homozygous *Dars1*^*D*367*Y*/*D*367*Y*^ mice and wildtype controls (Figure 1D). Explorative behavior, locomotor activity and anxiety were tested in the open field paradigm. Total distance traveled as well as distance traveled in the inner compartment of the apparatus were unchanged between groups (Figures 1E,F). The acoustic startle response (ASR), a reflex resulting in muscular activity in response to an acoustic stimulus, was determined to test *Dars1*^*D*367*Y*/*D*367*Y*^ mice for more subtle changes in information processing speed. Homozygous *mutants*, exposed to short sound pulses (40 ms) with increasing intensities ranging from 60 to 120 dB SPL, showed a significantly lower ASR compared to wildtype controls from 105 dB SPL to 120 dB SPL (sound levels 620 to 740; Figure 1G). The pre-pulse inhibition (PPI) measures sensorimotor gating mechanisms, and an impairment is an indicator for attentional processing deficits (Koch, 1999). We had observed PPI deficits in heterozygous *Dars1*-null mice (Fröhlich et al., 2017), however, *Dars1*^*D*367*Y*/*D*367*Y*^ mice were normal (Figure 1H).

### Aminoacylation Activity of Mutant AspRS

**Figure 2:**
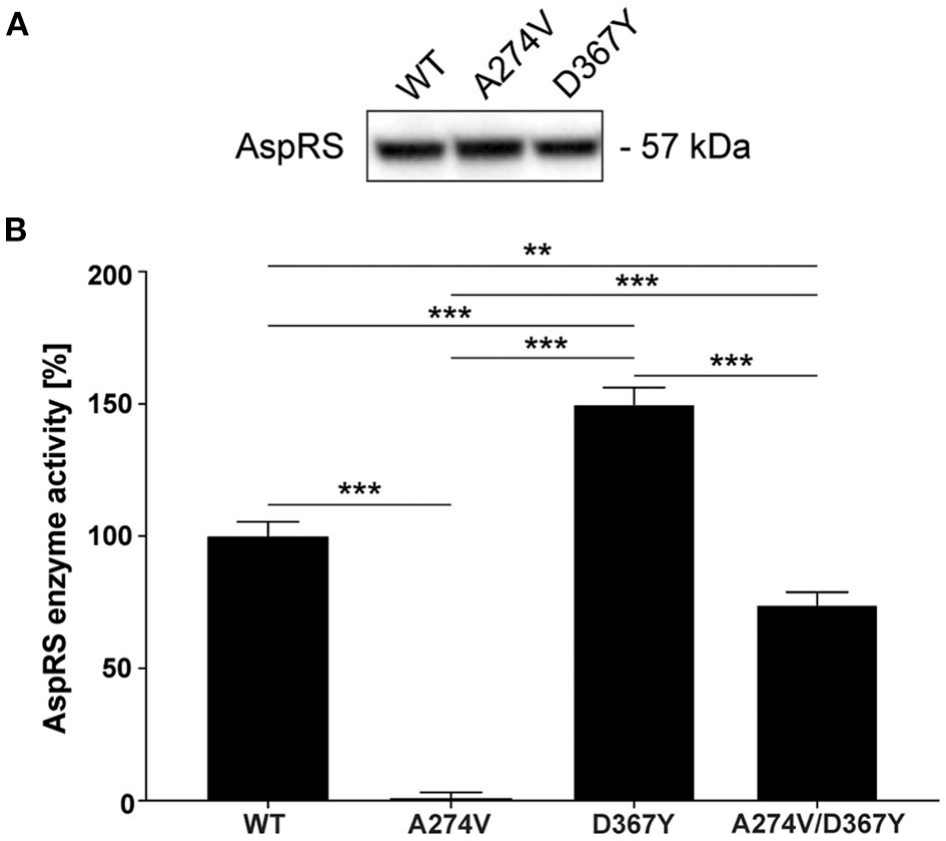
Aminoacylation activity of wildtype AspRS compared to AspRS carrying the A274V or D367Y mutations. **(A)** Western-blot depicting comparable expression levels of DARS1^WT^, DARS1^A274V^, DARS1^D367Y^ in HEK293 cell lysates. **(B)** Enzyme activity was measured using lysates of HEK293 cells transiently expressing DARS1^WT^, DARS1^A274V^, DARS1^D367Y^, or an equimolar mix of DARS1^A274V^ and DARS1^D367Y^. Cell lysates were incubated with *E. coli* tRNA, [^13^C_4_, ^15^N]-aspartate and ATP followed by quantification of [^13^C_4_,^15^N]-aspartate using LC-MS/MS. Aminoacylation activity of AspRS variants is displayed as percentage of WT AspRS activity. Data represent mean ± SEM (**p < 0.01, ***p < 0.001; One-way ANOVA).

The HBSL index patient was compound heterozygous for the A274V and D367Y *DARS1* mutations. Both mutations are located in the catalytic domain of the AspRS enzyme (Taft et al., 2013) indicating a direct impact on enzymatic activity. Therefore, we measured aminoacylation activity in lysates of HEK293 cells transiently expressing *DARS1*^*WT*^, *DARS1*^*A*274*V*^, *DARS1*^*D*367*Y*^, or an equimolar mix of *DARS1*^*A*274*V*^ and *DARS1*^*D*367*Y*^. Aminoacylation activity of mutant AspRS variants was normalized to the aminoacylation activity of wildtype AspRS (Figure 2). A higher value indicates more efficient charging of tRNA^Asp^ with aspartate. Strikingly, the A274V mutation completely abolished aminoacylation activity of AspRS, resembling a functional null mutation. In contrast, the D367Y mutation led to a slight increase in enzymatic activity. The enzyme activity measured in lysates of HEK293 cells transiently expressing a mix of *DARS1*^*A*274*V*^ and *DARS1*^*D*367*Y*^, which corresponds to the enzyme configuration of the index patient, ranged between the activities measured for *DARS1*^*A*274*V*^ and *DARS1*^*D*367*Y*^ alone (about 70% of wildtype AspRS activity).

### Compound Heterozygous *Dars1^*D*367*Y*/−^* Mice Display Developmental Deficits

We next crossed homozygous *Dars1*^*D*367*Y*/*D*367*Y*^ mice with heterozygous *Dars1*-null mice to create compound heterozygous *Dars1*^*D*367*Y*/−^ mice. Since the A274V mutation completely abolished AspRS activity, *Dars1*^*D*367*Y*/−^ mice can be considered a phenocopy of the compound heterozygous index patient (*DARS1*^*A*274*V*/*D*367*Y*^) in regard to enzyme activity.

**Figure 3:**
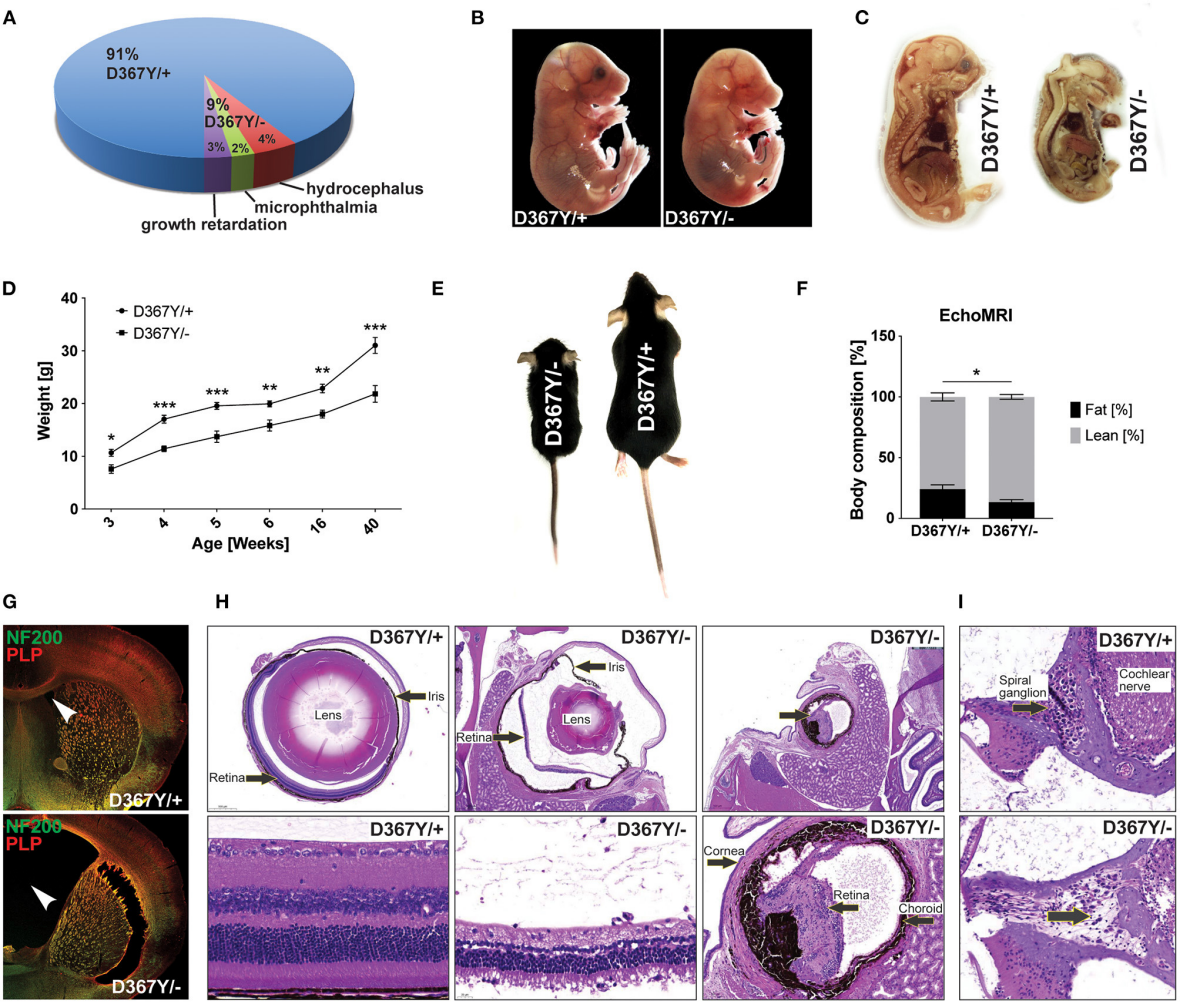
Compound heterozygous Dars1^D367Y/−^ mice display developmental deficits. **(A)** Genotype and phenotype distribution in the F1 generation resulting from breeding of homozygous D367Y and heterozygous Dars1-null mice. Of the 286 born mice (72 litters), only 25 (9%) were compound heterozygous (D367Y/–; expected: 50%). 48% of the born D367Y/– mice (4% of total mice) developed hydrocephalus during the first weeks of their life; 24% of D367Y/– mice (2% of total mice) had microphthalmia in 1 or 2 eyes. **(B)** At embryonic day 18 (E18), 57% of the embryos present *in utero* were D367Y/– (*n* = 21), however, they were significantly underdeveloped at this stage. **(C)** Sagittal sections of D367Y/+ and D367Y/– embryos at E18 showing the severe underdevelopment at this stage. **(D)** Weight of D367Y/+ and D367Y/– females (*n* = 4–11). **(E)** D367Y/– mice are smaller and lighter compared to age- and sex-matched D367Y/+ littermates. **(F)** EchoMRI body composition analysis revealed a significant reduction of body fat in D367Y/– mice (*n* = 8). **(G)** Coronal brain sections of 3-week-old D367Y/– mice with hydrocephalus and D367Y/+ control mice. Sections were labeled with NF200 (green) and PLP (red). **(H)** H&E staining of cross sections of the eyes showing various degrees of microphthalmia and retinal degeneration/atrophy in D367Y/– mice compared to D367Y/+ controls. **(I)** H&E staining of cross sections of the inner ear indicates reduced density of spiral ganglion cells (lower panel, arrow). Data represent mean ± SEM (*p < 0.05, **p < 0.01, ***p < 0.001; Two-way ANOVA).

The expected Mendelian ratio of the *Dars1*^*D*367*Y*/−^ and *Dars1*^*D*367*Y*/+^ offspring in the F1 generation is 50:50. However, of 286 born mice (72 litters), only 25 mice (9%) had the genotype *Dars1*^*D*367*Y*/−^. This developmental disadvantage was sustained postnatally as 48% of *Dars1*^*D*367*Y*/−^ mice (4% of all mice) developed hydrocephalus during the first 3 weeks of life and either died naturally or had to be euthanized (Figures 3A,G). 24% of *Dars1*^*D*367*Y*/−^ (2% of all littermates) suffered from microphthalmia or retinal degeneration in one or both eyes (Figures 3A,H). The remainder of the born *Dars1*^*D*367*Y*/−^ mice (28% of *Dars1*^*D*367*Y*/−^ mice or 3% of total mice) showed growth retardation and were significantly smaller and lighter compared to *Dars1*^*D*367*Y*/+^ littermates (Figures 3A,D,E). Bodyweight of *Dars1*^*D*367*Y*/−^ mice remained lower throughout life (Figure 3D) and EchoMRI body composition analysis revealed a significant reduction in body fat mass of *Dars1*^*D*367*Y*/−^ mice (13.5% body fat) compared to *Dars1*^*D*367*Y*/+^ littermates (24.3% body fat; Figure 3F). Lastly, H&E staining of cross sections of the inner ear showed a reduced density of spiral ganglion cells in adult *Dars1*^*D*367*Y*/−^ mice (Figure 3I).

Intriguingly, at embryonic day 18 (E18), 57% of the embryos present *in utero* were *Dars1*^*D*367*Y*/−^ (*n* = 21), which is in line with the expected Mendelian ratio when crossing homozygous *Dars1*^*D*367*Y*/*D*367*Y*^ mice with heterozygous *Dars1*-null mice. However, these embryos were severely underdeveloped at this stage (Figures 3B,C).

### Compound Heterozygous *Dars1^*D*367*Y*/−^* Mice Develop Late Onset Motor Deficits

**Figure 4:**
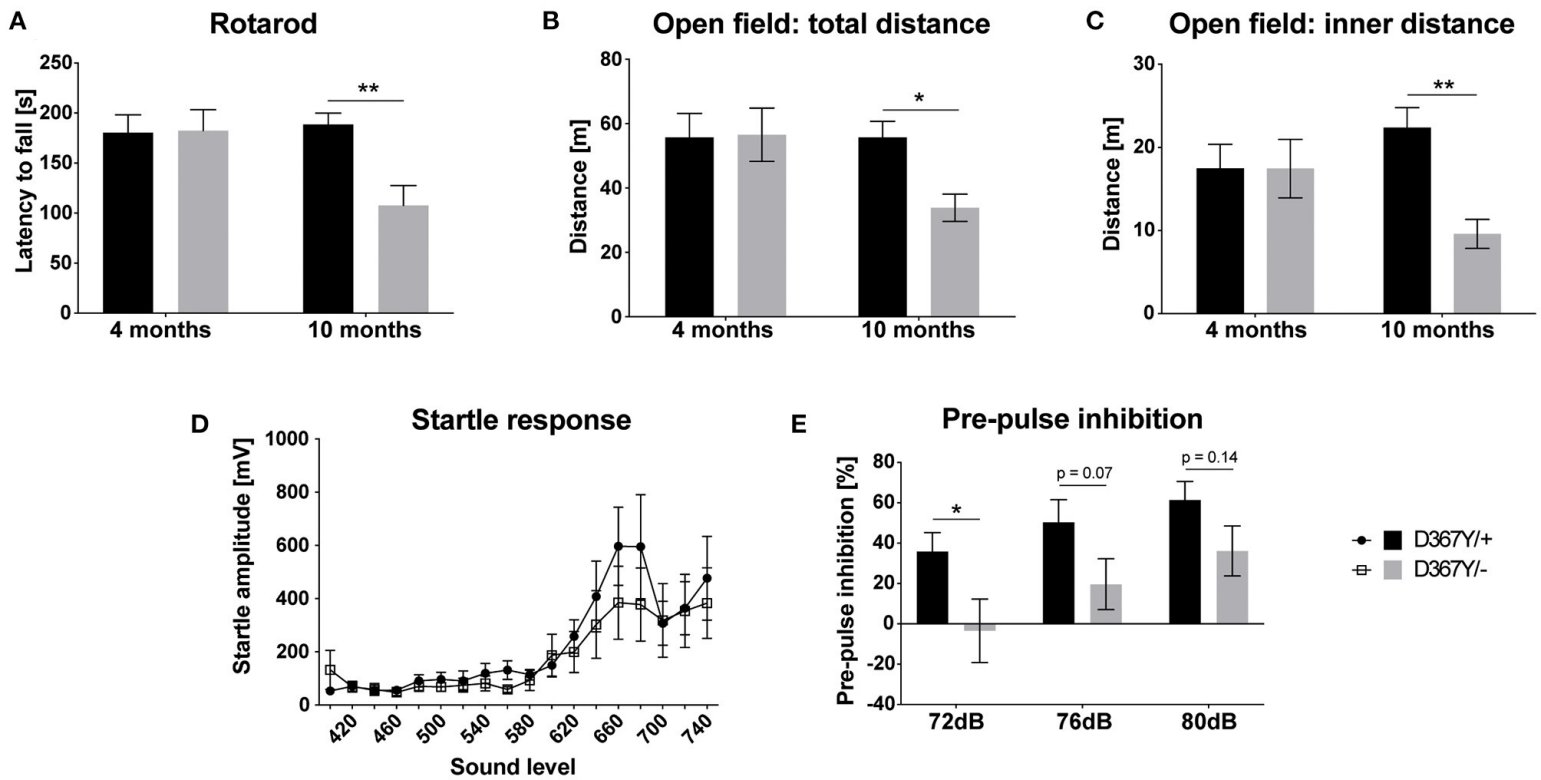
Compound heterozygous *Dars1*^*D*367*Y*/−^ mice show impaired pre-pulse inhibition and develop late onset motor deficits. **(A)** Motor coordination assessed by the rotarod test was unchanged at 4 months but was impaired at 10 months in D367Y/– mice compared to D367Y/+ controls (*n* = 7–10). **(B,C)** Total distance traveled in an open field test apparatus [**(B)**; *n* = 7–10] and distance in the inner compartment of the apparatus [**(C)**; *n* = 7–10]. No changes were observed at 4 months but at 10 months total distance and distance in the inner compartment were significantly reduced. **(D)** Four-months-old mice were exposed to sound pulses with increasing intensities (60–120 dB SPL, 40 ms) and the acoustic startle response was measured. No significant differences were observed between D367Y/– and D367Y/+ mice (*n* = 8). **(E)** Pre-pulse inhibition (72, 76, or 80 dB SPL pre-pulse 100 ms before the 120 dB SPL startle pulse) was reduced in 4-months-old D367Y/– mice (*n* = 8). Data represent mean ± SEM (*p < 0.05, **p < 0.01; Two-way ANOVA).

Despite the developmental delay and high mortality of *Dars1*^*D*367*Y*/−^ mice, once they passed early adolescence, *Dars1*^*D*367*Y*/−^ mice were phenotypically inconspicuous for most of their adult life. A hallmark of HBSL are motor deficits including severe leg spasticity and gait abnormalities. To see whether *Dars1*^*D*367*Y*/−^ mice develop similar motor deficits, we performed locomotor tests at two different timepoints (four and 10 months) to distinguish between early and late onset motor deficits. Motor coordination assessed by the rotarod test was unchanged at 4 months but was impaired at 10 months in *Dars1*^*D*367*Y*/−^ mice compared to *Dars1*^*D*367*Y*/+^ littermates (Figure 4A). Total distance traveled in an open field test apparatus and distance in the inner compartment of the apparatus were unchanged at 4 months of age (Figures 4B,C). At 10 months, however, the total distance and the distance in the inner compartment were significantly lower in *Dars1*^*D*367*Y*/−^ mice compared to *Dars1*^*D*367*Y*/+^ littermates indicating a reduction in locomotor activity and explorative behavior (Figures 4B,C). No significant differences in the ASR were observed between *Dars1*^*D*367*Y*/−^ and *Dars1*^*D*367*Y*/+^ mice (Figure 4D). PPI, on the other hand, was significantly lower in *Dars1*^*D*367*Y*/−^ mice following the 72 dB SPL pre-pulse and showed a trend toward a reduction for the 76 dB SPL and 80 dB SPL pre-pulses (Figure 4E).

### Spinal Cord Abnormalities in *Dars1^*D*367*Y*/−^* Mice

**Figure 5:**
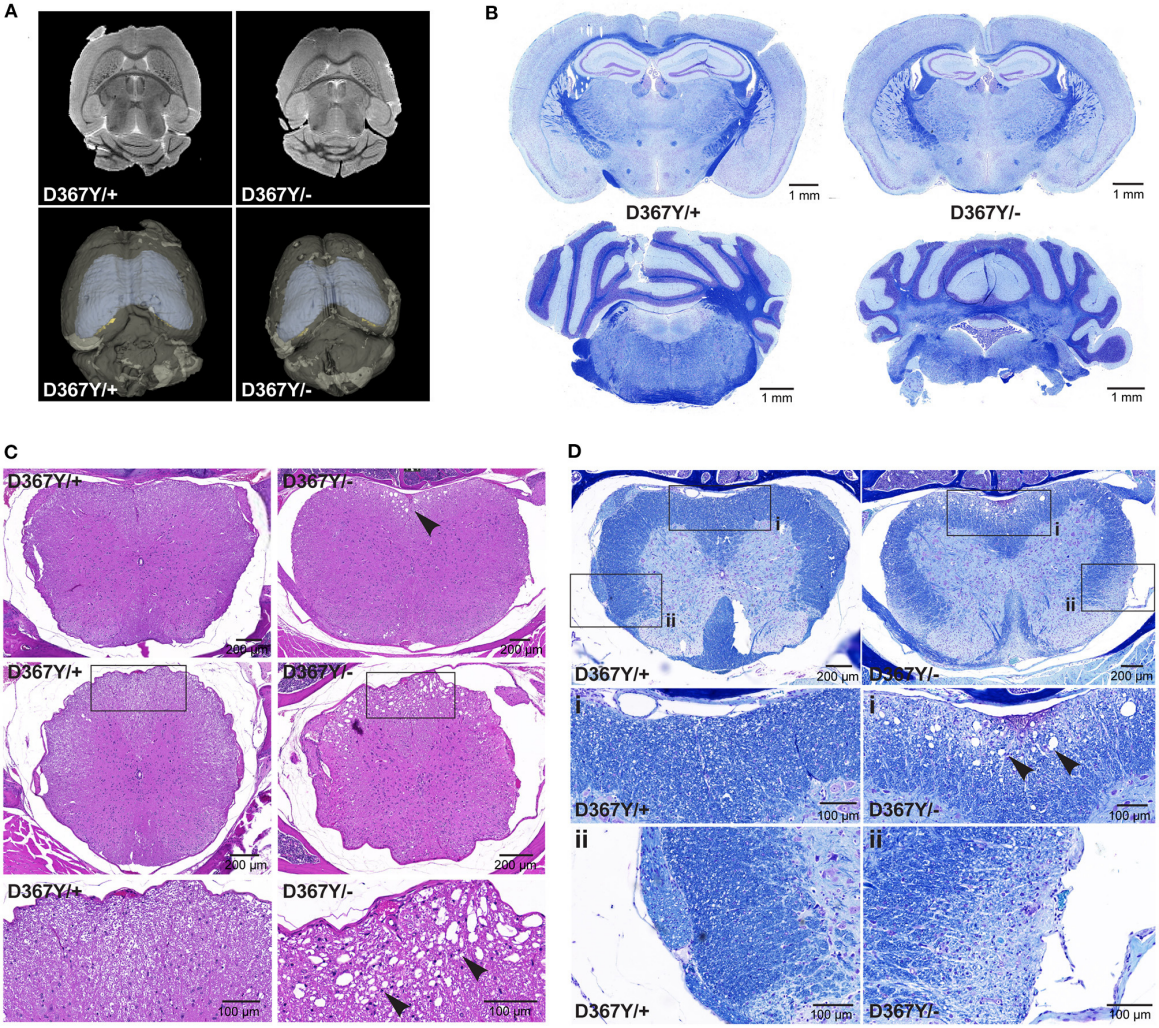
Compound heterozygous *Dars1*^*D*367*Y*/−^ mice display spinal cord white matter abnormalities. **(A)** Brain magnetic resonance imaging (MRI) revealed an overall smaller brain size of D367Y/– mice but did not show any other abnormalities or demyelination. **(B)** Luxol fast blue (LFB) staining of coronal brain sections revealed no morphological or myelination differences between genotypes. **(C)** Hematoxylin and Eosin (H&E) staining of spinal cord cross sections (top row: lumbar; middle row: thoracic) revealed severe vacuolization of the ventral white matter in D367Y/– mice (arrowheads; *n* = 2). **(D)** LFB staining of lumbar spinal cord cross sections confirmed white matter vacuolization in D367Y/– mice [close-up **(A)**, arrowheads] and revealed significant demyelination of the lateral and dorsal white matter [close-up **(B)**; *n* = 2]. Analyses were performed in 10-months-old mice.

In order to determine whether the behavioral deficits were accompanied by neurological changes, we analyzed central nervous system (CNS) integrity and myelination. Brain MRI revealed a smaller brain size of *Dars1*^*D*367*Y*/−^ mice but did not show any other overt abnormalities or demyelination (Figure 5A). LFB staining of coronal brain sections revealed intact brain morphology and no myelination differences between genotypes (Figure 5B). In the spinal cord, however, severe vacuolization of the ventral white matter was detected in 10-months-old *Dars1*^*D*367*Y*/−^ mice (Figure 5C) but not in *Dars1*^*D*367*Y*/+^ littermates. LFB staining confirmed white matter vacuolization in *Dars1*^*D*367*Y*/−^ mice (Figure 5D). In addition, aged *Dars1*^*D*367*Y*/−^ mice displayed severe demyelination of the lateral and dorsal white matter (Figure 5D).

### Reduced Expression of Major Myelin Proteins in *Dars1^*D*367*Y*/−^* Mice

**Figure 6:**
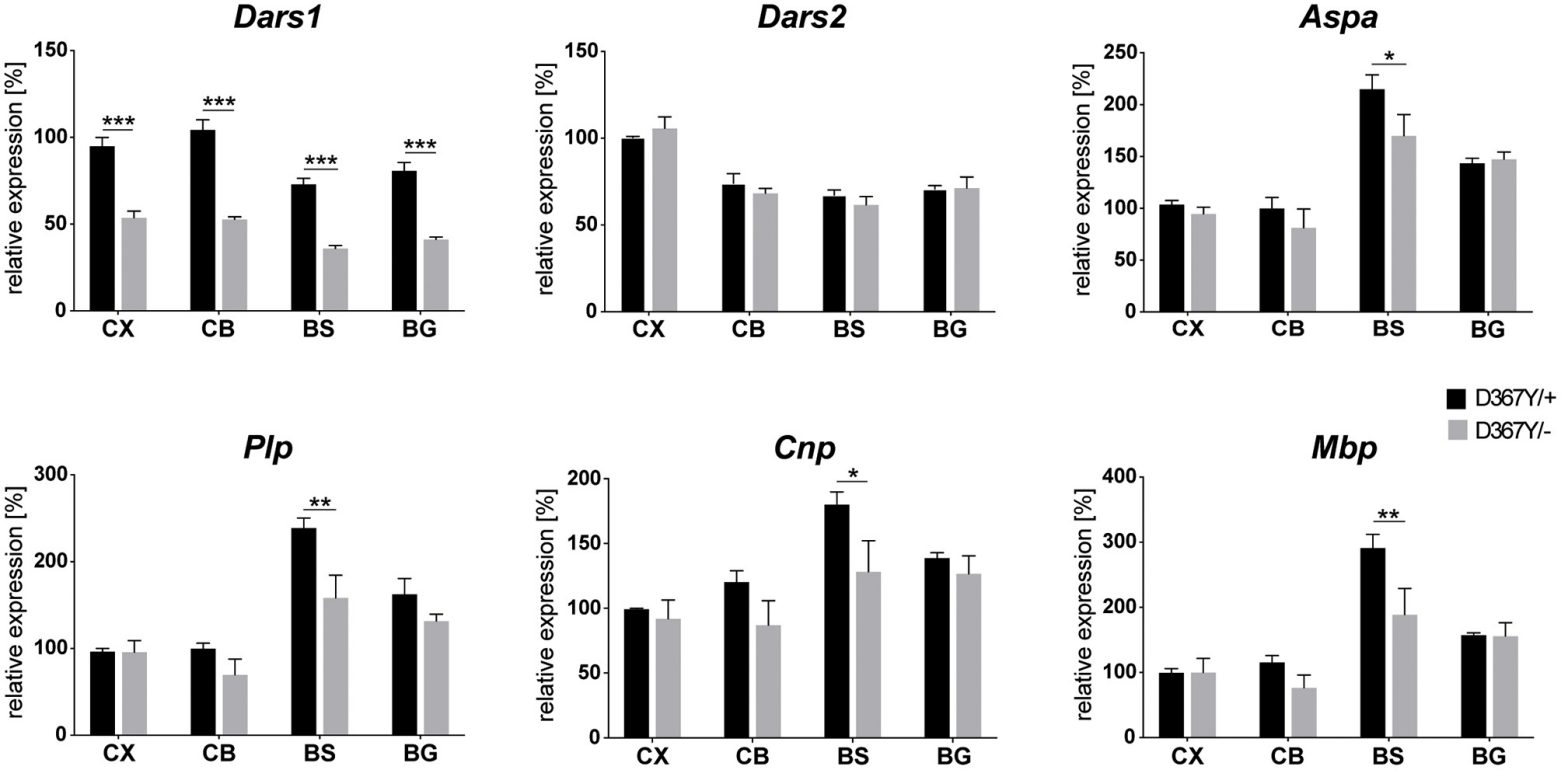
mRNA levels of Dars1 and the major myelin proteins are reduced in compound heterozygous *Dars1*^*D*367*Y*/−^ mice. mRNA was extracted from brain tissue of 10-month-old D367Y/– and D367Y/+ mice and quantified via qRT-PCR using TaqMan probes specific for Dars1, Dars2, Aspa, Plp, Cnp, and Mbp. Brain regions analyzed included cortex (CX), cerebellum (CB), brainstem (BS), and basal ganglia (BG). Expression levels were normalized to the housekeeper GusB (*n* = 4). Data represent mean ± SEM (*p < 0.05, **p < 0.01, ***p < 0.001; Two-way ANOVA).

**Figure 7:**
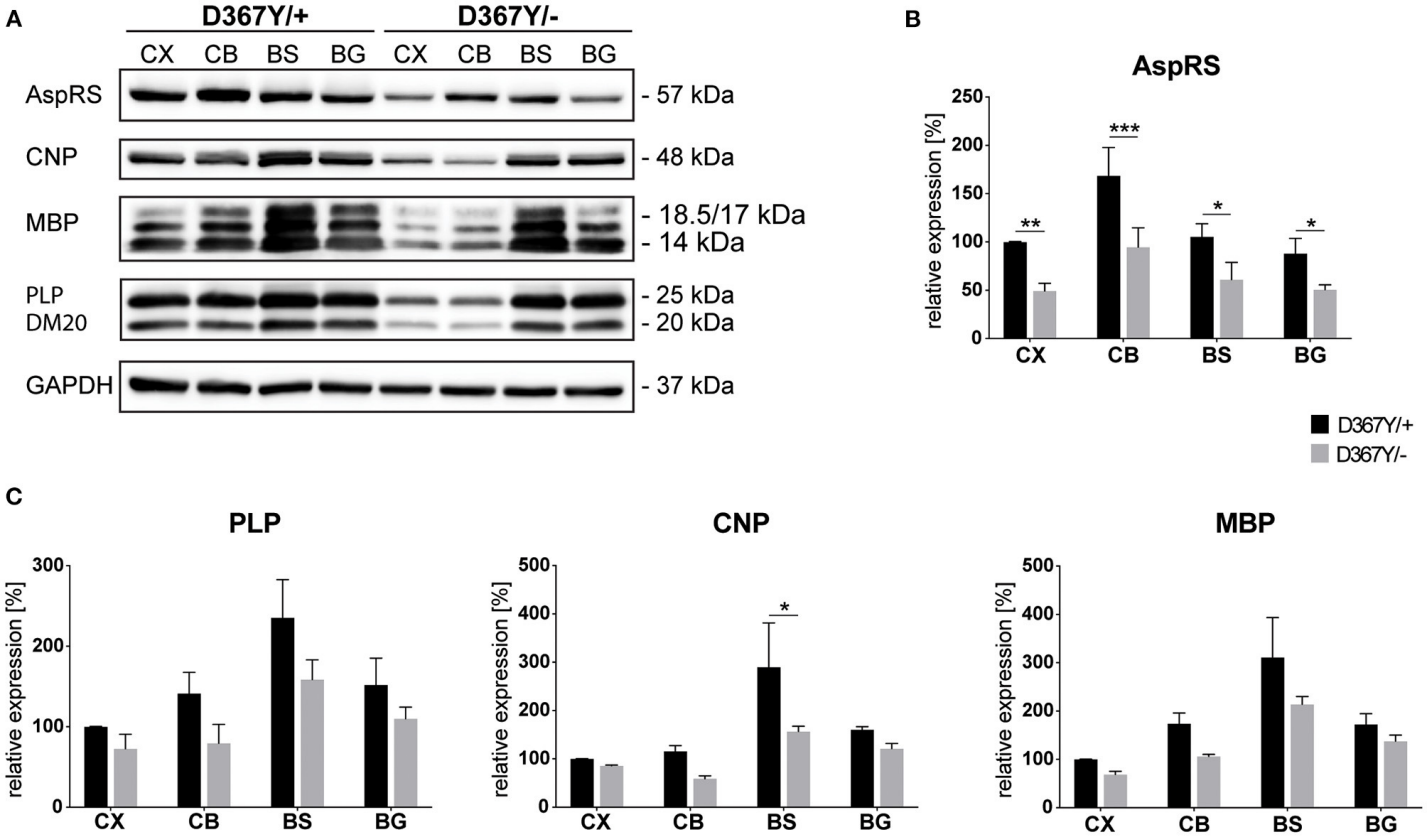
Expression levels of AspRS and the major myelin proteins in compound heterozygous *Dars1*^*D*367*Y*/−^ mice. **(A)** Western-blot depicting AspRS, the myelin proteins CNP, MBP, and PLP/DM20, as well as the housekeeping protein GAPDH in different brain regions of 10-month-old D367Y/– and D367Y/+ mice. Brain regions include cortex (CX), cerebellum (CB), brainstem (BS), and basal ganglia (BG). **(B)** Relative AspRS expression normalized to GAPDH (*n* = 3). **(C)** Relative expression of the myelin proteins PLP, CNP, and MBP normalized to GAPDH (*n* = 3). Data represent mean ± SEM (*p < 0.05, **p < 0.01, ***p < 0.001; Two-way ANOVA).

To determine whether myelin defects could be corroborated by reduced expression levels of the major myelin proteins in *Dars1*^*D*367*Y*/−^ mice, we analyzed brain tissue of 10-month-old *Dars1*^*D*367*Y*/−^ and *Dars1*^*D*367*Y*/+^ mice. The brain regions analyzed included cortex, cerebellum, brainstem and basal ganglia. First, we confirmed reduction of *Dars1* mRNA and AspRS protein in *Dars1*^*D*367*Y*/−^ mice. As expected, *Dars1* message and AspRS levels were reduced by 50% compared to *Dars1*^*D*367*Y*/+^ mice (Figure 6 top left and Figures 7A,B). mRNA levels of the mitochondrial counterpart *Dars2* were unchanged ruling out potential compensatory mechanisms (Figure 6 top middle). Markers of oligodendrocytes (Aspa) or myelin (PLP, CNP and MBP) were reduced at the mRNA (Figure 6) and protein (Figure 7) level, in cerebellum and brainstem. While this trend reached statistical significance only in the brainstem, this finding suggests potential demyelination or oligodendrocyte loss in the hindbrain of *Dars1*^*D*367*Y*/−^ mice.

## Discussion

To date, accurate HBSL models have been lacking, precluding examination of the underlying pathomechanism and testing of potential therapies. Here, we introduced the HBSL-causing *Dars1*^*D*367*Y*^ point mutation into the mouse genome. Unexpectedly, mice carrying this mutation homozygously did not show any characteristic HBSL pathology. To enhance the phenotype of *Dars1*^*D*367*Y*/*D*367*Y*^ mice, we crossed them with *Dars1-null* carriers. The *Dars1*^*D*367*Y*/−^ offspring displayed a severe developmental delay associated with early lethality either *in utero* or as a result of hydrocephalus during the first 3 weeks of life. Another frequently observed feature of *Dars1*^*D*367*Y*/−^ mice was microphthalmia in one or both eyes. All *Dars1*^*D*367*Y*/−^ mice were underdeveloped with reduced body weight and fat mass compared to *Dars1*^*D*367*Y*/+^ littermates but remained otherwise phenotypically inconspicuous until 10 months of age when they developed late onset motor deficits as well as vacuolization and demyelination of the spinal cord white matter. These behavioral and morphological changes were accompanied by reduced expression levels of various myelin and oligodendrocyte markers, predominantly in the hindbrain of *Dars1*^*D*367*Y*/−^ mice.

The A274V mutation resulted in a complete loss of aminoacylation activity, resembling a functional null mutation. In light of the lethal phenotype of homozygous null mice (Fröhlich et al., 2017) this suggests that a bi-allelic A274V mutation would not be viable in mouse or man. Intriguingly, the D367Y mutation did not decrease AspRS enzyme activity but instead led to a slight increase in activity. A potential explanation for the observed increase in enzymatic activity of *Dars1*^*D*367*Y*^ could be reduced specificity of AspRS for tRNA^Asp^. As a consequence, other tRNAs would bind to AspRS and subsequently be mischarged with aspartate. These mischarged tRNAs would then incorporate the wrong amino acid into the growing polypeptide chain during translation ultimately resulting in either non-functional proteins or unfolded and misfolded proteins. An accumulation of un- and misfolded proteins in the endoplasmic reticulum (ER) can trigger the unfolded protein response (UPR), an adaptive pathway that under normal circumstances protects the cell from ER stress and restores normal ER function (Lin and Popko, 2009). If the UPR fails to cope with sustained ER stress, apoptosis is triggered to eliminate malfunctioning cells (Faitova et al., 2006; Szegezdi et al., 2006). This normally protective mechanism can become pathological and has been implicated in the pathophysiology of many neurodegenerative (Szegezdi et al., 2006) and white matter diseases such as Charcot-Marie-Tooth disease, Pelizaeus-Merzbacher disease, Vanishing White Matter disease, and multiple sclerosis (Lin and Popko, 2009). A similar underlying pathophysiological mechanism has been suggested for HBSL (Fröhlich et al., 2017) and is supported by data from this study. Accordingly, activation of the mitochondrial unfolded protein response (UPR^mt^) has been demonstrated in conditional *Dars2* knockout mice (Dogan et al., 2014; Aradjanski et al., 2017). *Dars2* encodes the mitochondrial aspartyl-tRNA synthetase (mt-AspRS) and mutations in the *Dars2* gene result in *l*eukoencephalopathy with *b*rainstem and *s*pinal cord involvement and *l*actate elevation (LBSL), which was first described in 2003 (van der Knaap et al., 2003; Scheper et al., 2007).

Patients affected by HBSL or LBSL display similar symptomology and MRI patterns involving the same CNS tracts and structures. While both diseases might share a common underlying disease mechanism, such as failure of the UPR and UPR^mt^ due to an excessive accumulation of un- and misfolded proteins, there is no functional redundancy between AspRS and mt-AspRS. This has been demonstrated in *Dars1* and *Dars2* null mice, both displaying early embryonic lethality (Dogan et al., 2014; Fröhlich et al., 2017). HBSL and LBSL mainly affect the nervous system, a phenomenon that has also been observed in other ARS-related conditions (Fuchs et al., 2019). The majority of reported LBSL patients possess a splice site mutation in intron two of the *DARS2* gene. This splice defect particularly affects neural cells providing an explanation for the CNS predilection seen in LBSL (van Berge et al., 2012). The selective vulnerability of neural cells in HBSL can be explained with the distinct AspRS expression pattern in the CNS of mice and humans (Fröhlich et al., 2017, Fröhlich et al., 2018). Accordingly, we observed CNS defects in *Dars1*^*D*367*Y*/−^ mice including vacuolization and demyelination of the spinal cord white matter. White matter vacuolization is the most common myelin pathology and has been described for many leukodystrophies including Canavan's disease and Vanishing white matter disease (Duncan and Radcliff, 2016). When vacuolization occurs in conjunction with myelin degeneration it can be referred to as spongiform degeneration, however, myelin vacuolization can also occur independent of demyelination (Duncan and Radcliff, 2016). In the case of *Dars1*^*D*367*Y*/−^ mice, the vacuolization in the spinal cord was accompanied by demyelination and might reflect a separation of myelin layers (Barkovich, 2000).

Protein synthesis is often regarded as a house-keeping function of all cells. Nevertheless, many aspects of protein synthesis are differently regulated across cell types and developmental stages in order to establish differences in cell identity, function and homeostasis (Buszczak et al., 2014). These differences might explain why *Dars1*^*D*367*Y*/−^ mice are particularly susceptible to changes in protein synthesis during development resulting in developmental delay and in some cases premature death. Once *Dars1*^*D*367*Y*/−^ mice made it through adolescence, the remaining AspRS activity seems to be sufficient to maintain normal tissue homeostasis. However, the effects of impaired protein synthesis seem to either accumulate over the lifetime or become more profound in aged mice, resulting in the observed late onset motor deficits and spinal cord abnormalities.

Many *Dars1*^*D*367*Y*/−^ mice developed hydrocephalus during the first 3 weeks of life. Despite not being a frequently observed feature of HBSL, it has been described in one HBSL patient who consequently required ventriculo-peritoneal shunting to release pressure on the brain (Ong et al., 2020). Another commonly detected symptom of *Dars1*^*D*367*Y*/−^ mice was severe microphthalmia or even anophthalmia, major structural malformations or even complete absence of the eye. These abnormalities were not only present in the born *Dars1*^*D*367*Y*/−^ mice but also visible in 18-day-old *Dars1*^*D*367*Y*/−^ embryos indicating that they occur early during embryogenesis as a result of impaired protein synthesis. The peripheral pathologies observed in *Dars1*^*D*367*Y*/−^ mice, such as reduced size, body weight and fat mass as well as microphthalmia have not been reported for HBSL patients yet. While this might be due to the low number of cases reported to date, tests for peripheral deficits should be included in the clinical context in the future.

Homozygous *Dars1*^*D*367*Y*/*D*367*Y*^ mice showed a lower ASR compared to wildtype mice, whereas *Dars1*^*D*367*Y*/−^ mice displayed reduced PPI of the ASR. A PPI reduction is indicative of impaired sensorimotor gating mechanisms (Koch, 1999). The genetic cause of this impairment appears to be the *Dars1*-null mutation since reduced PPI was also a feature of heterozygous *Dars1*-null mice (Fröhlich et al., 2017). The ASR is mediated by a simple neuronal circuit of the lower brainstem involving neurons of the caudal pontine reticular nucleus (PnC) (Koch, 1999). PPI, on the other hand, involves a complex interplay of many brain areas ultimately resulting in a dampening of the ASR via the PnC. PPI is reduced in a variety of neurological disorders including schizophrenia, Huntington's disease, Tourette's syndrome, and ADHD (Swerdlow and Geyer, 1998; Koch, 1999) and, based on our results, might also be a feature of HBSL. Reduced PPI was present in severely affected *Dars1*^*D*367*Y*/−^ mice as well as in mildly affected heterozygous *Dars1*-null mice (Fröhlich et al., 2017), suggesting to test for ASR and PPI in the clinical context as they might support an early diagnosis of HBSL.

While *Dars1*^*D*367*Y*/−^ mice do not model every aspect of the clinical HBSL picture, they enable studies of both, early and late onset deficits, which so far was precluded in *Dars1* knockout mice due to embryonic lethality. As such, mice harboring the hypomorphic *Dars1*^*D*367*Y*^ allele represent the first tool enabling therapeutic proof-of-concept studies including nutraceutical L-ornithine-L-aspartate (LOLA) supplementation to boost AspRS activity (Das et al., 2020) or *DARS1* gene replacement therapy. The introduction of other HBSL point mutations might produce a more accurate disease model with an early disease onset as seen in the majority of HBSL patients. Moreover, the use of a conditional knockout system will be instrumental in dissecting the contribution of *Dars1*-deficiency in select organs or cell lineages similar to what has been reported for *Dars2* (Aradjanski et al., 2017; Nemeth et al., 2020; Rumyantseva et al., 2020).

## Data Availability Statement

The original contributions presented in the study are included in the article/supplementary materials, further inquiries can be directed to the corresponding author/s.

## Ethics Statement

The animal study was reviewed and approved by University of New South Wales Animal Care and Ethics Committee.

## Author Contributions

DF and MK designed the study. DF, MIM, AJK, MJH, GSS, and AB conducted the research. DF, MIM, and MK analyzed the data. GDH contributed to the experimental design and manuscript preparation. DF and MK led the project and the manuscript production. All authors read and approved the final manuscript.

## Conflict of Interest

MK is an employee of Boehringer Ingelheim Pharma GmbH & Co. KG. The remaining authors declare that the research was conducted in the absence of any commercial or financial relationships that could be construed as a potential conflict of interest.
